# Imaging diagnosis in peripheral nerve injury

**DOI:** 10.3389/fneur.2023.1250808

**Published:** 2023-09-14

**Authors:** Yanzhao Dong, Ahmad Alhaskawi, Haiying Zhou, Xiaodi Zou, Zhenfeng Liu, Sohaib Hasan Abdullah Ezzi, Vishnu Goutham Kota, Mohamed Hasan Abdulla Hasan Abdulla, Alenikova Olga, Sahar Ahmed Abdalbary, Yongsheng Chi, Hui Lu

**Affiliations:** ^1^Department of Orthopedics, The First Affiliated Hospital, Zhejiang University, Hangzhou, Zhejiang Province, China; ^2^Department of Orthopedics, The Second Affiliated Hospital of Zhejiang Chinese Medical University, Hangzhou, Zhejiang Province, China; ^3^PET Center, The First Affiliated Hospital, College of Medicine, Zhejiang University, Hangzhou, Zhejiang Province, China; ^4^Department of Orthopedics, Third Xiangya Hospital, Central South University, Changsha, Hunan Province, China; ^5^Zhejiang University School of Medicine, Hangzhou, Zhejiang Province, China; ^6^Department of Neurology, Republican Research and Clinical Center of Neurology and Neurosurgery, Minsk, Belarus; ^7^Department of Orthopedic Physical Therapy, Faculty of Physical Therapy, Nahda University, Beni Suef, Egypt; ^8^The Intensive Care Unit of Huzhou Traditional Chinese Medicine Hospital, Huzhou, Zhejiang Province, China; ^9^Alibaba-Zhejiang University Joint Research Center of Future Digital Healthcare, Zhejiang University, Hangzhou, Zhejiang Province, China

**Keywords:** peripheral nerve injury, imaging, ultrasound, magnetic resonance imaging, positron emission tomography

## Abstract

Peripheral nerve injuries (PNIs) can be caused by various factors, ranging from penetrating injury to compression, stretch and ischemia, and can result in a range of clinical manifestations. Therapeutic interventions can vary depending on the severity, site, and cause of the injury. Imaging plays a crucial role in the precise orientation and planning of surgical interventions, as well as in monitoring the progression of the injury and evaluating treatment outcomes. PNIs can be categorized based on severity into neurapraxia, axonotmesis, and neurotmesis. While PNIs are more common in upper limbs, the localization of the injured site can be challenging. Currently, a variety of imaging modalities including ultrasound (US), computed tomography (CT) and magnetic resonance imaging (MRI) and positron emission tomography (PET) have been applied in detection and diagnosis of PNIs, and the imaging efficiency and accuracy many vary based on the nature of injuries and severity. This article provides an overview of the causes, severity, and clinical manifestations of PNIs and highlights the role of imaging in their management.

## Introduction

**Figure 1:**
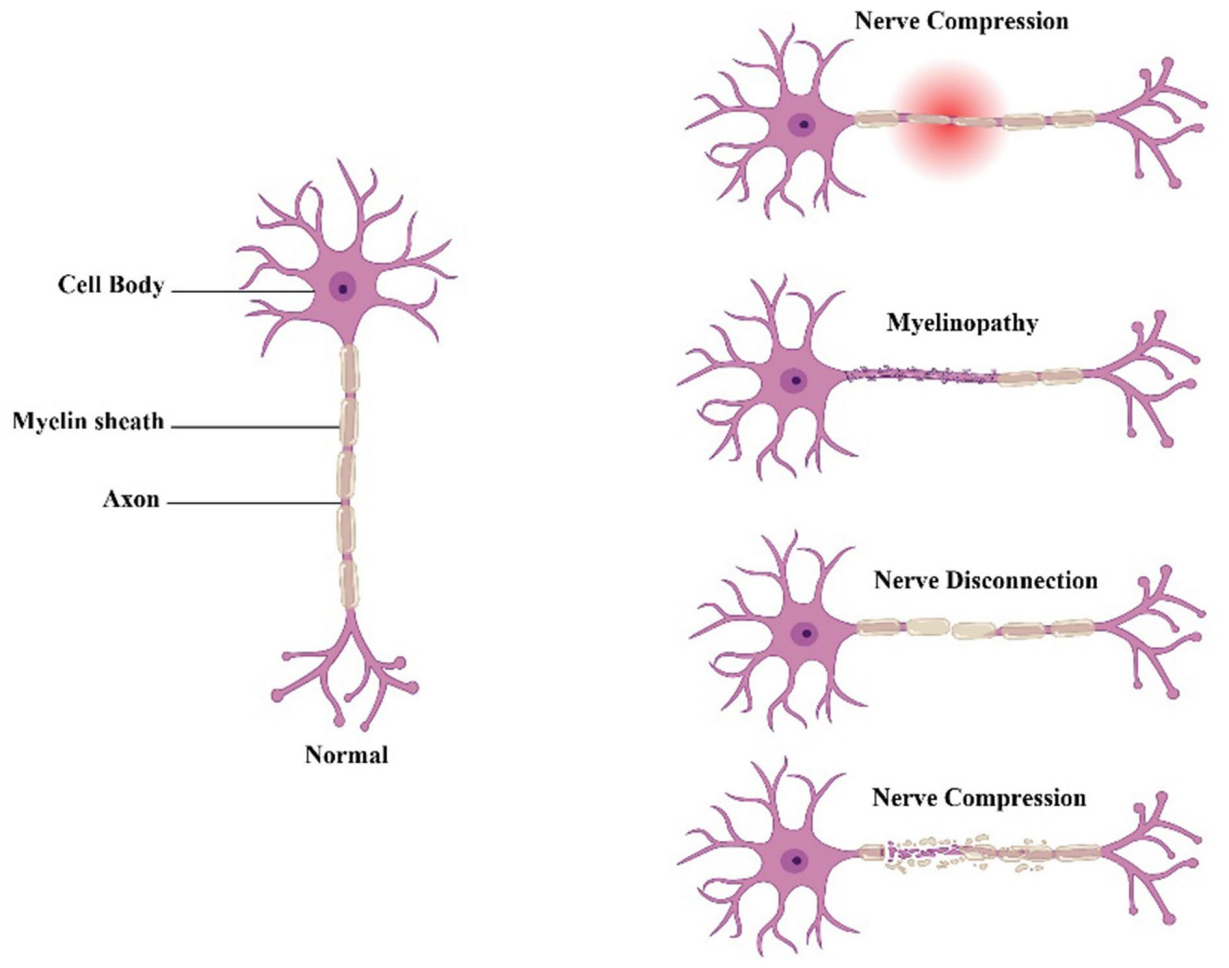
Neuropathic pain caused by different types of peripheral nerve damage.

Peripheral nerve injury (PNI) refers to any damage or trauma to the nerves located outside the central nervous system, such as those in the limbs or face. This can be caused by a variety of factors, including physical trauma, compression, inflammation, or disease. PNI can result in a range of symptoms, including pain, weakness, numbness, and loss of function (1). Peripheral nerves are complex structures that serve as conduits for relaying information between the brain and other body parts. They consist of axons, which are long, thin fibers that carry electrical impulses and supporting cells known as Schwann cells. These cells wrap around the axons to form a myelin sheath in myelinated nereves, which acts as an insulator and helps to speed up signal transmission (2). When a peripheral nerve is injured, the axons can be damaged or severed, disrupting the flow of signals between the brain and the affected area of the body. In addition, the Schwann cells can also be damaged, which can further impede nerve function and delay the healing process. There are several types of PNI, each with its own specific symptoms and treatment options. The types of PNI include compression injuries; these occur when a nerve is compressed or pinched, often as a result of repetitive motions or sustained pressure (Figure 1). Examples of compression injuries include carpal tunnel syndrome, which affects the median nerve in the wrist, and ulnar nerve entrapment, which affects the nerve that passes across on the interior of the elbow of the elbow. Stretch injuries occur when a nerve is stretched beyond its normal range of motion, often as a result of a sudden impact or overextension. Stretch injuries can cause damage to the nerve fibers and the myelin sheath, leading to symptoms such as pain, weakness, and numbness. Also, crush injuries are where the nerve is compressed or squeezed, often as a result of a direct blow or trauma. Crush injuries can cause severe damage to the nerve fibers and the surrounding tissue, leading to symptoms such as loss of sensation and motor function. Moreover, transection injuries occur when a nerve is completely severed, often as a result of a sharp object or traumatic injury. Transection injuries require immediate medical attention and always require surgical intervention to repair the damaged nerve (3–5). The symptoms of PNI can differ depending on the severity and type of the injury. Common symptoms include pain, numbness, tingling, weakness, and loss of motor function. In some cases, PNI can also cause changes in skin color or temperature, as well as muscle wasting or atrophy. Treatment options for PNI depend on the type and severity of the injury. Mild cases of PNI may be treated with rest, physical therapy, and over-the-counter pain medication. More severe cases may require surgical intervention, such as nerve grafting or nerve repair. In some cases, medication such as steroids or painkillers may also be prescribed to help manage symptoms (1). Recovery from PNI can be a slow and gradual process and may take several weeks or months. During this time, physical therapy and rehabilitation can be an important part of the recovery process, helping to restore function and improve overall mobility (6). In some cases, however, PNI can lead to permanent damage and long-term disability.

PNI are categorized into five groups by Sidney Sunderland as follows: Axon damage in grade 1 is indicated by denervation alterations on electromyography. Endoneurial tube injury, grade 2, is characterized by acute denervation alterations on the EMG. On an EMG, grade 3 is marked by a total destruction of the axon and endoneurial tubes. Grade 4 nerve disruption is characterized by an intact epineurial tube and persistent denervation alterations on the electromyogram (EMG). Complete nerve transection in grade 5 is indicated by no EMG contraction potentials (7).

In conclusion, peripheral nerve injury is a complex and potentially debilitating condition that can result from a variety of causes. Understanding the different types of PNI and their symptoms can help individuals recognize when they may be at risk for injury, and seek appropriate treatment as soon as possible. While recovery from PNI can be a slow and challenging process, with the right care and support, many individuals are able to regain function and mobility and return to their normal activities.

Radiological imaging is a crucial tool in the diagnosis of peripheral nerve injuries. MRI, ultrasound, and CT scans are all commonly used modalities in this process. Each of these imaging techniques provides unique benefits and can help identify the location and severity of nerve damage. In the following sections, we will provide a more detailed description of each imaging modality and how it is used in the diagnosis of peripheral nerve injuries.

### Ultrasound

Peripheral nerve injuries (PNIs) can vary greatly in causes, severity and clinical manifestations. Depending on the site, severity and cause, therapeutic intervention could range from conservative medication treatment to various surgeries, which would require precise orientation and planning to avoid further iatrogenic damage (8). Common causes of PNIs include penetrating injury, compression, stretch and ischemia (9), while causes including electric shock, thermal damage, infections and toxic/chemical damage are relatively rare (10, 11). Based on severity, PNIs can also be categorized into neurapraxia, axonotmesis, and neurotmesis, according to H. J. Seddon (12). If untreated, PNIs sometimes result from compression from surround tissues or abnormal nerve healing, as in carpal tunnel syndrome and traumatic neuromas, respectively. On the other hand, while studies have suggested that PNIs occur more often in upper limbs, especially in the median nerve, radial nerve and ulnar nerve (13, 14), localization of the injured site is challenging as physical examination is less accurate inpatients with less prominent symptoms.

**Figure 2:**
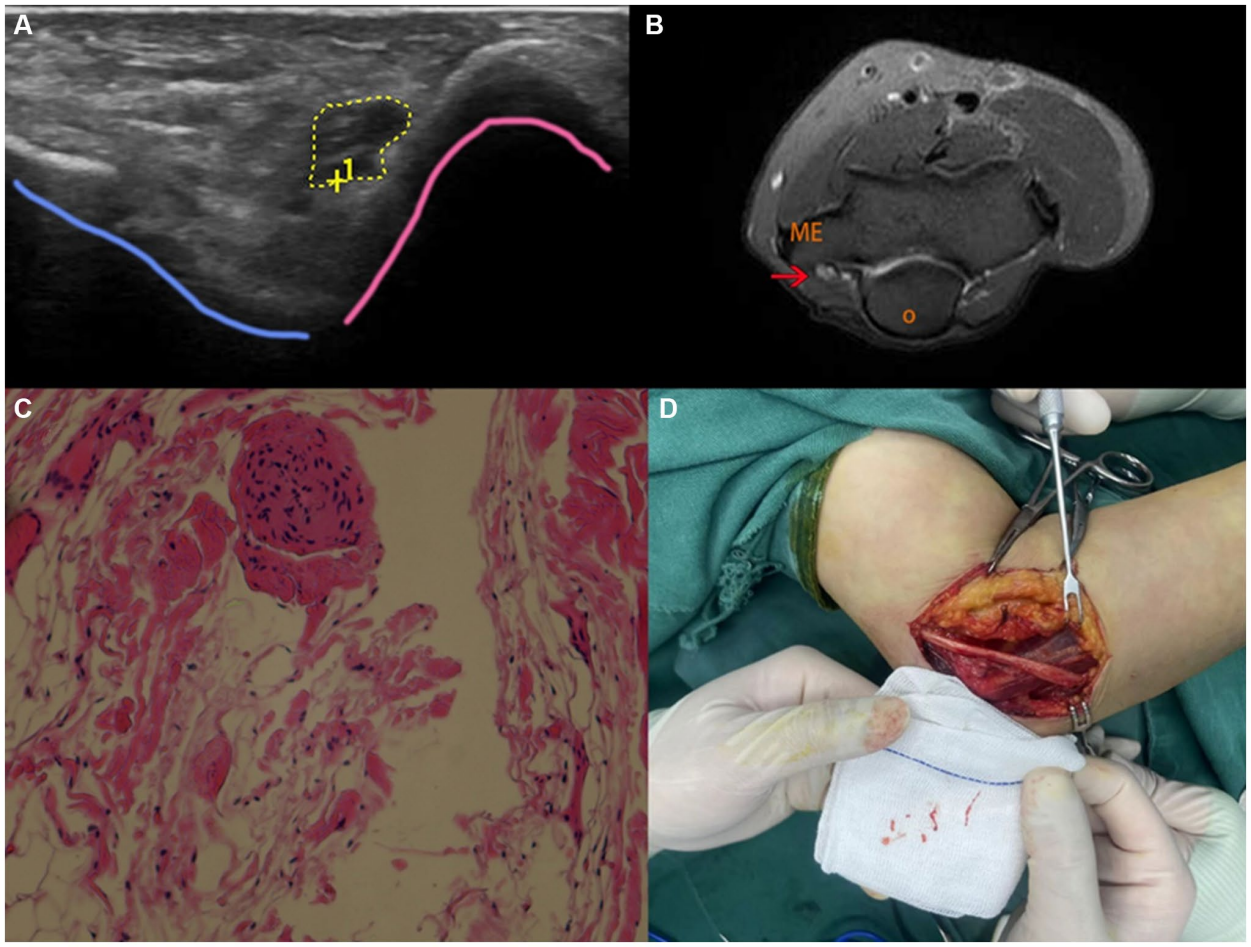
US, MRI, pathological examination and intraoperative images of a 54-year-old female patient suffering from numbness and tenderness on the ulnar side of left hand. **(A)** US imaging on the left elbow showed hypoechoic thickening of the nerve fascicles at the cubital tunnel; **(B)** MRI showed significant thickening of the left ulnar nerve at the cubital tunnel with increased T2-weighted signal intensity. Red arrow marks the ulnar nerve swelling. O (olecranon), ME (medial epicondyle). **(C)** Pathological examination indicated small piece of fibrofatty tissue; **(D)** intraoperative image showed adhesion and edema of the ulnar nerve and surrounding tissue.

Ultrasound (US) is a safe and time-effective non-invasive imaging modality often applied in the diagnosis and management of peripheral nerve injuries. Compared with magnetic resonance imaging (MRI), US is not only capable of depicting nerve images in real-time, but more affordable as well. One of the reasons for applying US in peripheral nerve injuries is that peripheral nerve injuries majorly occur at relatively superficial sites (Figure 2), which could be captured in high-resolution by ultrasonography with high-frequency probes (15). In two clinical studies, C. Cokluk and K. Aydin investigated the efficacy of US in evaluating PNIs in 36 patients with upper extremity PNIs and 22 patients with lower extremity PNIs (16, 17). Their findings suggest that US can be used to assess the extent of the injury, determine if the nerve is completely or incompletely severed, detect the presence of hematoma or foreign bodies, evaluate nerve stumps and perilesional scar tissue, and identify the presence of neuromas. Additionally, Cokluk et al. conducted another study in 14 patients with penetrating PNIs ranging from glass cut to civilian gunshot, where US was applied intraoperatively adjacent to the injured site (8). Particularly in pediatrics, when patients and peripheral nerves are smaller, ultrasound is particularly suited for the examination of peripheral nerves. An advantage over MRI is that no anesthesia or intravenous contrast is necessary. Orthopedic implants and bullet fragments, which pose problems for MRI, may not always be a problem for ultrasonography. For a comprehensive assessment of several nerves, muscle denervation, and deeper tissues, MRI is better suited. Decision-making is aided by both ultrasound and MRI, which play complementary roles in the evaluation of nerve damage (18). In comparison with electro-diagnostics, US not only identified nerve injuries but also accurately localized the injuries, determined their severity, identified the presence of neuromas or stumps, and detected the formation of perineural scar tissue pre- and intraoperatively. Additionally, High-frequency probes used in contemporary ultrasound devices In order to obtain (semi)-quantitative assessments of the many histological components of the peripheral nerve, probes and equipment enable a “real-time dissection” of the nerve. Using highly sensitive color/power Doppler imaging, ultrasound can also enable the observation of tiny vascular structures with sluggish blood flow. As a result, ultrasonography can offer a precise visual representation of the structure of superficial nerves including nerve fascicles, interfascicular epineurium and epifascicular epineurium (19). Depending on the stage of injury and the particular kind of neuropathy, the perfusion pattern of the peripheral nerve varies greatly. High-sensitive color/power Doppler imaging, on the other hand, can be used to visualize tiny vascular structures with sluggish blood flow and can precisely measure the peripheral nerve’s microcirculation in both normal and pathological circumstances. As a result, the peripheral nerve’s perfusion pattern might be thought of as a fascinating diagnostic component, particularly when healing from damage with axonotmesis, and sonographic follow-up can be helpful in this regard (19). By combining steady-state/fast imaging with steady-state (CISS/FIESTA) MRI, Shaye et al. discovered that preoperative high-resolution neurovascular imaging was associated with higher rates of surgical success in cases of medically intractable TN (20).

In addition to penetrating injuries and foreign objects, chronic compression from surrounding tissues is a frequent cause of PNIs, with CTS being the most prominent disorder. The carpal bones and transverse carpal ligament (TCL) collectively constitute a restricted osteofibrous canal, allowing the median nerve and digital flexor tendons to traverse from the forearm into the hand. Congenital anomalies, extended manual labor, inflammation, and tissue edema may lead to nerve compression, initially causing sporadic nocturnal paresthesia and dysesthesias. These symptoms may then escalate in frequency and advance to anesthesia and thenar muscle atrophy due to extensive axonal degeneration (21, 22). Although the symptoms of CTS are distinctive, certain studies have indicated that ultrasound (US) can identify patients with unfavorable surgical outcomes resulting from anatomical predisposition (23–26). In these cases, US-guided acupotomy could be a potential alternative. A recent study conducted by Zhou et al. compared the effectiveness of ultrasound-guided acupuncture and conventional acupuncture in loosening the TCL in 100 cadaveric upper limb specimens (27). The study found that the use of ultrasound guidance in acupuncture significantly reduced the incidence of injury to blood vessels and tendons compared to the conventional method. However, the incidence of median nerve injury was found to be similar in both methods. These findings suggest that ultrasound guidance may be a safer alternative to conventional acupuncture for loosening TCL, particularly in cases where there is a higher risk of injury to nearby structures. Recent evidence suggests that neuromuscular ultrasound (NMUS) is emerging as an important tool for the diagnosis and management of peripheral nerve disorders. NMUS enables real-time visualization of neural structures and provides critical information about nerve structure and function to complement clinical examination and electrodiagnostic studies. Examples of dynamic NMUS techniques used to evaluate and monitor peripheral neuropathy include: (1) Identification of underlying etiology in tarsal tunnel syndrome using threshold values for tibial nerve cross-sectional area within the tarsal tunnel. (2) Elucidation of etiologic factors in proximal tibial neuropathies by ultrasound. (3) Application of the Bochum ultrasound score, which assesses ulnar nerve cross-sectional area at Guyon’s canal and the arm, radial nerve area at the spiral groove, and sural nerve area in the calf. (4) Quantitative measures of intraneural blood flow, such as manual Doppler signal counts or Doppler waveform analysis to determine blood flow velocity. (5) Contrast-enhanced ultrasound and measurement of maximum perfusion intensity for accurate characterization of intraneural vascularity (28).

In conclusion, ultrasound is an increasingly valuable imaging tool for diagnosing and managing peripheral nerve injuries (PNIs). Its real-time capability and high resolution provide safe, cost-effective scans that help assess injury extent and nerve severance. In both the acute and chronic phases of peripheral neuropathy, (semi)-quantitative measures of the peripheral nerve can be combined with various sonographic patterns of its histological components. Therefore, acute and chronic compression of the peripheral nerve may be distinguished using high-resolution ultrasound imaging (19). Ultrasound has paved the way for innovative treatment options, improving patient outcomes and quality of life in cases of PNIs.

### Computed tomography (CT)

CT scan is mainly useful for identifying bony abnormalities that may be contributing to nerve injuries, such as bone spurs, fractures, or joint dislocations. CT scans use X-rays to create detailed images of the bones and other radiopaque tissues in the body, making them an excellent tool for identifying these types of structural abnormalities (29, 30). In some cases, CT imaging may be used as an initial screening tool for peripheral nerve injuries, particularly if there is suspected involvement of nearby bones or joints (31) and CT scans can be used to locate locations where nerves are compressed due to conditions including tumors, herniated discs, or bone spurs. CT scans can be a useful tool for identifying the potential causes of PNI by making the structures close to the nerves visible (32). However, CT imaging is less useful for visualizing radiolucnettissues such as nerves, as they do not show up well on traditional CT scans. In order to visualize nerves more clearly, a specialized type of CT scan called CT myelography may be used. This involves injecting a contrast dye into the spinal fluid, which then fills the nerve sheaths and allows for better visualization of the nerves on the CT scan (33, 34).

While CT myelography can be helpful for identifying nerve injuries, it is not without risks. The injection of contrast dye carries a small risk of allergic reactions or other adverse effects, and the procedure can be uncomfortable for some patients. Additionally, CT myelography exposes patients to ionizing radiation, which can increase the risk of cancer over time (33).

Overall, CT imaging can be a valuable tool for diagnosing peripheral nerve injuries, particularly when used in conjunction with other imaging modalities such as MRI and ultrasound. While it may not be the first choice for visualizing nerves, it can provide important information about bony abnormalities that may be contributing to nerve injury. As with any medical imaging test, the decision to use CT imaging for diagnosing peripheral nerve injuries should be made on a case-by-case basis, taking into account the patient’s individual needs and medical history.

### Magnetic resonance imaging

**Figure 3:**
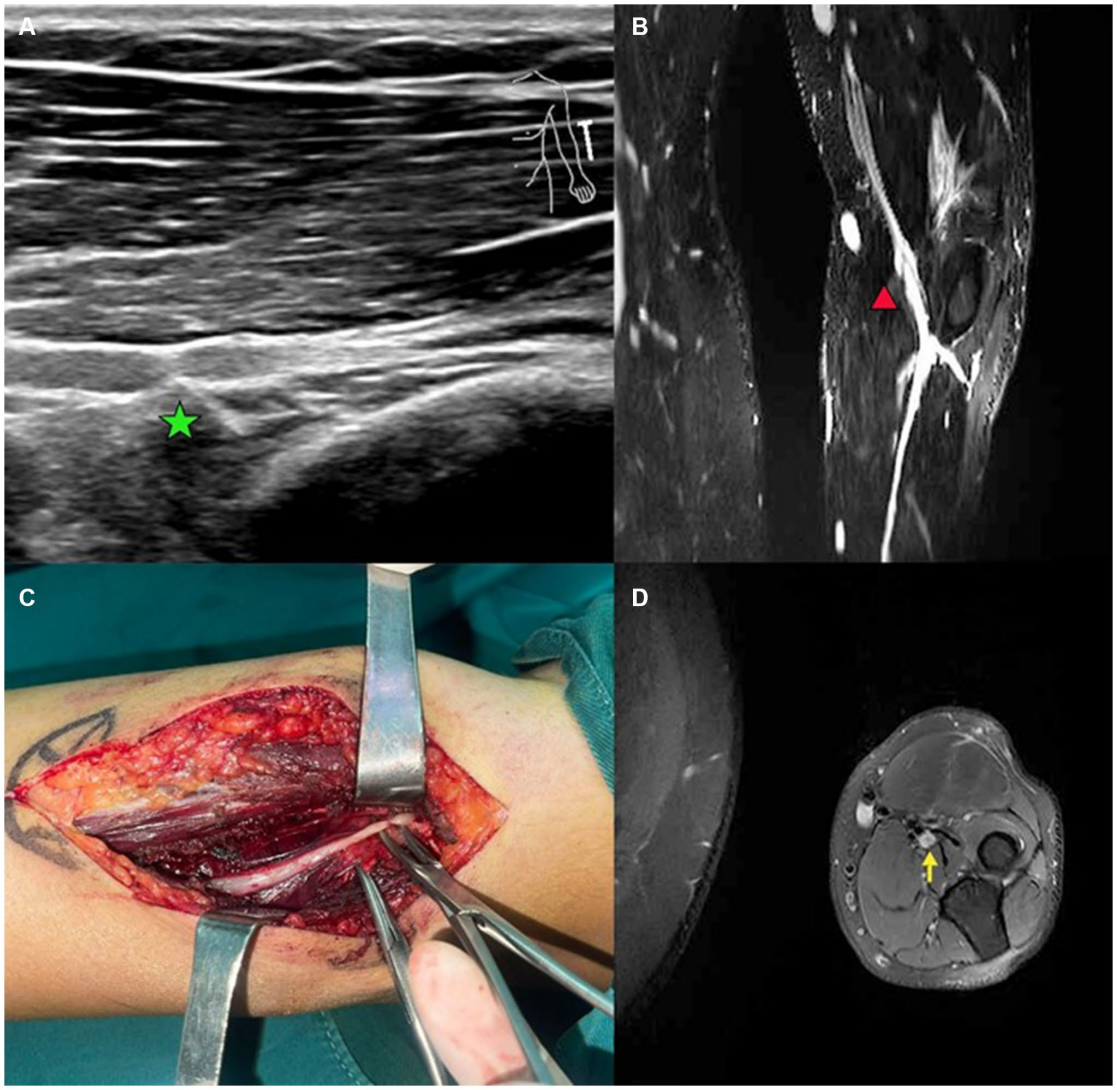
A case of patient with radial nerve entrapment. **(A)** US imaging of the radial nerve. Green star marks the proximal site of segmental entrapment. **(B)** Functional MRI of the radial nerve. Red triangle marks the site of radial nerve swelling. **(C)** Intraoperative image of deep branch of radial nerve. Segmental entrapment is observed. **(D)** MRI image of entrapped radial nerve. Yellow arrow makes the radial nerve swelling.

Magnetic resonance imaging (MRI) is a non-invasive imaging based diagnostic tool that can be employed to detect peripheral nerve injury (35, 36). High magnetic field MRI imaging can be utilized to execute high contrast neurography with the help of fat suppression sequences and this kind of imaging modality enables us to observe structural connectivity through the use of diffusion tensor imaging and tractography (35). Peripheral nerve MRI can reveal pathological changes such as nerve compression, inflammation, or tumors in peripheral nerves and other associated components of the peripheral nervous system (Figure 3) (36).

MRI can help diagnose peripheral nerve injury by providing detailed images of the nerves and surrounding tissues. It can identify the location and extent of nerve damage, which can help guide treatment decisions. For example, if a patient has carpal tunnel syndrome, MRI can show whether there is compression of the median nerve in the wrist (35). In addition to diagnosis, MRI can also be used to monitor the progression of nerve injuries over time (36). some researches indicate The nerve lesion is clearly revealed by MR-neurography, which also aids in determining whether surgery is necessary and the best course of action (37, 38). In a cohort study, the paper also supports this view. It suggests that for roughly 4 weeks within the first 90 days following the trauma, MR-neurography has a considerable time-saving effect on decision-making. This could aid in overcoming the paradigm of “watch and wait” tactics used in the first three to six months following a peripheral nerve injury (39).

The benefits of using MRI for diagnosing peripheral nerve injury include its non-invasive nature and its ability to provide detailed images of soft tissues. Unlike other diagnostic tools such as electromyography (EMG), which involves inserting needles into muscles to measure electrical activity, MRI does not require any invasive procedures (35). Additionally, MRI can provide information about the entire length of a nerve, whereas EMG only measures activity at specific points along a nerve (36). This makes MRI a valuable tool for diagnosing complex cases where multiple nerves may be affected.

Magnetic resonance imaging (MRI) can reveal pathological changes in peripheral nerves (35, 36, 40–45). Affected nerves and associated structures show changes in both morphology and signal intensity when observed with the assistance of magnetic resonance imaging (45). MRI can also help us visualize in great detail, nerve lesions in areas that are normally difficult to localize with the help of diagnostic modalities such as computed tomography or ultrasound imaging owing to superior contrast and resolution (45). Magnetic resonance neurography is a specialized magnetic resonance based technique that has already been proven to help evaluate, characterize and diagnose traumatic and compressive lesions which affect the peripheral nerves, their nerve roots, and the nerve plexii (46, 47).

Coming to focal neuropathies, irrespective of whether they are traumatic or have been caused as a result of nerve entrapment, Magnetic resonance neurography has helped improve the diagnostic accuracy in a significant manner by allowing us to directly visualize the underlying pathological changes that accompany such neuropathies. This technique has also enabled the first *in vivo* visualization of neuronal degeneration and nerve regeneration and associated changes (46). Next, when we consider, demyelinating neuropathies, an intraneural T2WI signal increase was seen upon conducting an MRI examination (46). However, the hyperintensity observed on the T2WI appeared to be restricted to the lesion side of the injury without any proximal or distal fascicles being seen (46).

Together with ultrasound, MRI has been instrumental in enabling the diagnosis and management of patients with peripheral neuropathies and associated ailments. Neurons are often compressed due to anatomical constrains as they generally travel through structures such as myofascial planes or confined spaces such as fibro-osseous or fibromuscular tunnels in the proximity of a joint. The enlargement of the contents of the such tunnels due to tumors, injuries, and associated oedemas and hematomas, or repetitive muscle contraction can lead to further compression of the nerve and related structures. The lesions associated with entrapment neuropathies usually present as non-enhancing fusiform lesions on MRI and there is also a flattening of the nerve at the site of entrapment (44).

According to a retrospective review of patients who underwent neuromuscular ultrasound, this imaging modality is more sensitive than magnetic resonance-based approaches. Additionally, ultrasound also shows equivalent specificity, and seems to better identify multifocal lesions than MRI based modalities (Table 1) (32). However, there is a caveat to this result in that, the study in question actually excluded conditions such as idiopathic carpal and cubital tunnel syndromes and when it comes to the comparison, the authors compared the accuracy of ultrasound and MRI for the detection of focal peripheral nerve pathologies in patients with mononeuropathies and brachial plexopathies alone (32). Ultrasound based imaging helped detect these lesions more frequently as compared to MRI based approaches and talking about specificity and exclusion, it was seen that ultrasound excluded nerve pathologies with equal accuracy as compared to MRI. Ultrasound imaging was also noted to be accurate in more patients (32).

**Table 1:** Comparison between US and MRI in terms of affordability, clinical application, image quality and suitability for various clinical settings.

Criteria	Ultrasound	MRI
Cost	Less expensive	More expensive
Accessibility	Widely available	Limited availability
Safety	Non-invasive and no radiation exposure	Non-invasive but exposure to magnetic field and radio waves
Imaging speed	Real-time imaging	Longer acquisition time
Image quality	Good for superficial structures, bones, and joints	Better for soft tissue contrast
Sensitivity	Can detect small nerve injuries	High sensitivity
Specificity	Less specific in determining the type of injury	More specific in determining the type of injury
Patient comfort	Comfortable and well-tolerated	Claustrophobic and noisy environment
Operator dependency	Highly operator-dependent	Less operator-dependent
Suitability for different types of injuries	Suitable for evaluating peripheral nerve entrapment and focal neuropathies	Suitable for evaluating more complex injuries, such as nerve root avulsion or plexus injuries

Both MRI and ultrasound are accurate methods in visualizing peripheral nerves (32). However, ultrasound is preferred as it has been shown to be more sensitive in detecting peripheral nerve pathology (32). Together, ultrasound and MRI are instrumental in facilitating diagnosis and management of patients with peripheral neuropathies (32).

Both MRI and ultrasound imaging have benefits and limitations in the diagnosis of peripheral nerve injuries. Ultrasound is more sensitive than MRI in detecting peripheral nerve pathology, has equivalent specificity, and better identifies multifocal lesions than MRI (32). However, ultrasound has limitations such as operator dependence, limited field of view, and difficulty visualizing deep-seated nerves or those surrounded by bone or air-filled structures (32). One of the most significant technical constraints of this diagnostic method is the “incorrect” interpretation of the ultrasonic artifacts. When the ultrasound beams strike the target structure at an angle other than perpendicular (oblique insonation angle), anisotropy artifact results. Because of this, certain sound waves may reflect but not return to the probe, rendering what would ordinarily appear to be a hyperechoic structure to appear hypo- or anechoic (48).

On the other hand, MRI provides high contrast neurography by fat suppression sequences and shows structural connectivity through the use of diffusion tensor imaging (32). It can reveal pathological changes in the peripheral nervous system such as nerve compression, inflammation or tumors (32). Moreover, it can provide information about the central nervous system that may not be visible on ultrasound. However, MRI has limitations such as being expensive compared to ultrasound and requiring patients to lie still for an extended period during scanning. Additionally, some patients may experience claustrophobia during an MRI scan (32).

### Positron emission tomography (Pet) imaging

**Figure 4:**
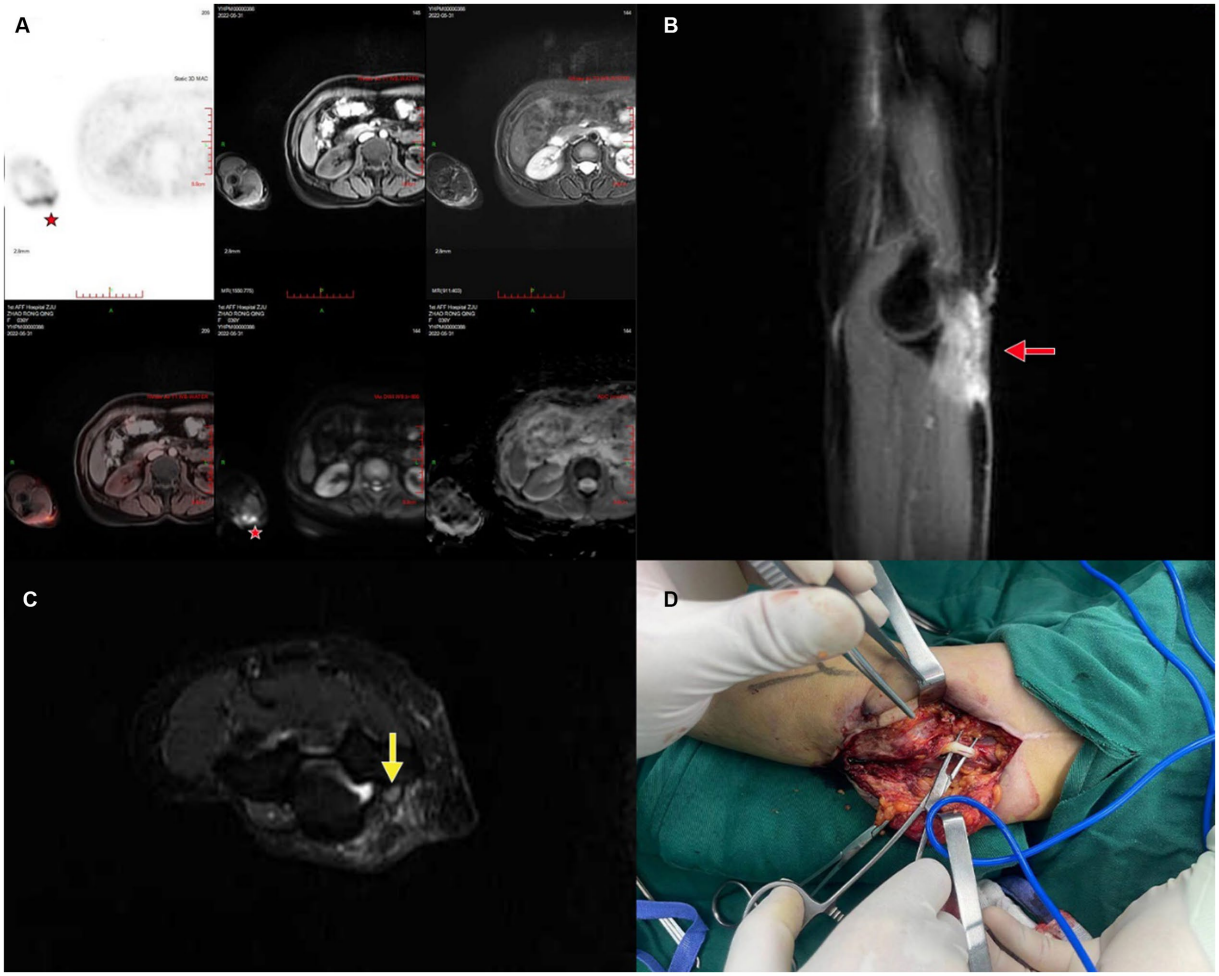
A case of patient suffering initially from allodynia and later a mass on the right elbow, which was pathologically confirmed as epithelioid sarcoma with ulnar nerve injury. **(A)** PET/CT showing the affected area; **(B)** coronal plane of the mass adjacent to ulnar nerve, red arrow marks the mass; **(C)** sagittal plane of the mass and the ulnar nerve, yellow arrow marks the swelling of the ulnar nerve; **(D)** intraoperative image of mass excision and nerve preservation.

As a primary symptom of PNIs, allodynia could occur without significant changes in nerve morphology, adding to difficulty for diagnosis by US or MRI. However, allodynia is generally associated with altered glucose metabolism, which can be accurately detected by PET imaging (Figure 4). In 2015, a study by Behera et al. observed increased 18F-FDG on PET/MRI in association with allodynia caused by PNIs in a small animal model, and concluded that allodynia could lead to increased cellular metabolism by sensitizing the sensory neurons and inducing nerve regeneration (49). On the other hand, compared with US or MRI, PET imaging detects molecular changes and therefore makes it possible to target certain signaling pathways in PNI. Endoplasmic reticulum sigma-1 receptor (S1R) is widely distributed in the nervous system and could bind to various ligands to exert neuroprotective and regenerative functions (50–53). Given that S1R could bind to a range of different proteins, Bin et al. designed a S1R-specific radioligand, 18F FTC-146, and observed accurate binding to sites of PNIs on PET/MRI, which was further confirmed by autoradiography and immunostaining (54).

Furthermore, the electrophysiology of PNIs is distinctly different, thus the necessity of electromyography (55). Nerve conduction *via* voltage-gated ion channels, however, can be measured differently using radioligands. In terms of pain perception and transmission in peripheral sensory nerve, a study in 2022 pointed out that 18F-radiocaine, a radio-labeled derivative of lidocaine, could be used for PET/MRI imaging of sciatic nerve ligation in rats, as observed by Nicole et al. not only was the uptake of 18F-radiocaine greater in the acute and chronic phase, but the uptake in nerve tissue is significantly higher than in muscle as well (56).

While PET imaging in PNIs is accurate, available with various ligands, and can detect in absence of significant morphological changes, it is still limited by side-effects associated with radiopharmaceutical, and relatively low affordability compared with electromyography, US and MRI.

## Conclusion

In summary, magnetic resonance imaging is an effective tool for diagnosing peripheral nerve injury. It provides detailed images of nerves and surrounding tissues without requiring any invasive procedures. The benefits of using MRI include its ability to identify the location and extent of nerve damage and its ability to monitor changes over time. Ultrasound has a lot of advantages in the diagnosis and treatment of peripheral nerves, like non-invasiveness, real-time imaging, cost-effectiveness, et al. As compared to other imaging examination methods, ultrasound has the unique strength of being real-time. It can provide real-time dynamic imaging to monitor surgical procedures and treatment outcomes in real time. In the diagnosis of peripheral nerves, MRI and ultrasound exhibit significant complementarity and can be combined to provide more comprehensive and accurate diagnosis. MRI provides high-resolution static imaging of peripheral nerves, while ultrasound provides real-time dynamic functional information. The integration of the two modalities can maximize the diagnostic level. Peripheral nerve imaging using high-resolution ultrasound and MRI is now possible with greater precision thanks to the development of higher frequency probes and enhanced MR field strength.

## Author contributions

HL and YC designed the study. YD and AA drafted the manuscript. HZ, XZ, and ZL performed literature selection and drew the figures. SE and VK collected patient data. SA, AO, and MA revised the manuscript. All authors contributed to the article and approved the submitted version.

## Funding

The study was funded by Zhejiang Provincial Natural Science Foundation Academic Exchange Program (grant number: LS21H0600010). The funding bodies had no role in the design of the study; in the collection, analysis, interpretation of data and in drafting the manuscript.

## Conflict of interest

The authors declare that the research was conducted in the absence of any commercial or financial relationships that could be construed as a potential conflict of interest.

## Publisher’s note

All claims expressed in this article are solely those of the authors and do not necessarily represent those of their affiliated organizations, or those of the publisher, the editors and the reviewers. Any product that may be evaluated in this article, or claim that may be made by its manufacturer, is not guaranteed or endorsed by the publisher.
